# Mid-Infrared High-Power InGaAsSb/AlGaInAsSb Multiple-Quantum-Well Laser Diodes Around 2.9 μm

**DOI:** 10.3390/nano15020139

**Published:** 2025-01-17

**Authors:** Hongguang Yu, Chengao Yang, Yihang Chen, Jianmei Shi, Juntian Cao, Zhengqi Geng, Zhiyuan Wang, Haoran Wen, Enquan Zhang, Yu Zhang, Hao Tan, Donghai Wu, Yingqiang Xu, Haiqiao Ni, Zhichuan Niu

**Affiliations:** 1 Key Laboratory of Optoelectronic Materials and Devices, Institute of Semiconductors, Chinese Academy of Sciences, Beijing 100083, China; ghy@semi.ac.cn (H.Y.); chenyihang18@semi.ac.cn (Y.C.); shijianmei@semi.ac.cn (J.S.); caojuntian@semi.ac.cn (J.C.); gengzhengqi@semi.ac.cn (Z.G.); wangzhiyuan@semi.ac.cn (Z.W.); zhangenquan2001@semi.ac.cn (E.Z.); zhangyu@semi.ac.cn (Y.Z.); dhwu@semi.ac.cn (D.W.); yingqxu@semi.ac.cn (Y.X.); nihq@semi.ac.cn (H.N.); 2 Center of Materials Science and Optoelectronics Engineering, University of Chinese Academy of Sciences, Beijing 100049, China; 3 International Quantum Academy, Shenzhen 518048, China; 18877116333@163.com (H.W.); tanhao@iqasz.cn (H.T.); 4 Hefei National Laboratory, Hefei 230088, China

**Keywords:** miscibility gap, interdiffusion process, molecular epitaxy beam, antimonide laser diodes, mid-infrared emitters

## Abstract

Antimonide laser diodes, with their high performance above room temperature, exhibit significant potential for widespread applications in the mid-infrared spectral region. However, the laser’s performance significantly degrades as the emission wavelength increases, primarily due to severe quantum-well hole leakage and significant non-radiative recombination. In this paper, we put up an active region with a high valence band offset and excellent crystalline quality with high luminescence to improve the laser’s performance. The miscibility gap of the InGaAsSb alloy was systematically investigated by calculating the critical temperatures based on the delta lattice parameter model. As the calculation results show, In_0.54_Ga_0.46_As_0.23_Sb_0.77_, with a compressive strain of 1.74%, used as the quantum well, is out of the miscibility gap with no spinodal decomposition. The quantum wells exhibit high crystalline quality, as evidenced by distinct satellite peaks in XRD curves with a full width at half maximum (FWHM) of 56 arcseconds for the zeroth-order peak, a smooth surface with a root mean square (RMS) roughness of 0.19 nm, room-temperature photoluminescence with high luminous efficiency and narrow FHWM of 35 meV, and well-defined interfaces. These attributes effectively suppress non-radiative recombination, thereby enhancing internal quantum efficiency in the antimonide laser. Furthermore, a novel epitaxial laser structure was designed to acquire low optical absorption loss by decreasing the optical confinement factor in the cladding layer and implementing gradient doping in the p-type cladding layer. The continuous-wave output power of 310 mW was obtained at an injection current of 4.6 A and a heatsink temperature of 15 °C from a 1500 × 100 μm^2^ single emitter. The external quantum efficiency of 53% was calculated with a slope efficiency of 0.226 W/A considering both of the uncoated facets. More importantly, the lasing wavelength of our laser exhibited a significant blue shift from 3.4 μm to 2.9 μm, which agrees with our calculated results when modeling the interdiffusion process in a quantum well. Therefore, the interdiffusion process must be considered for proper design and epitaxy to achieve mid-infrared high-power and high-efficiency antimonide laser diodes.

## 1. Introduction

Mid-infrared emitters are in great demand for numerous applications in the field of molecular spectroscopy [1], biomedical diagnostics [2], and industrial process control [3] owing to the prominent characteristic absorption lines of technological and greenhouse gases in the mid-infrared spectral range [4,5], such as NH_3_ (2.1 μm), HF (2.5 μm), and CH_4_ (2.35 μm and 3.3 μm). These emitters also attract much attention in LIDAR [6], free-space optical communications [7], and infrared countermeasures [8], due to consideration of the high transparency atmospheric transmission window in this spectrum. Additionally, these emitters are also utilized as pump light sources to enhance other emitters lasing at longer wavelengths and achieve efficient detection of light in other spectral bands [9,10]. Due to their compactness, scalability, high efficiency, and cost-effectiveness, semiconductor lasers are the dominant choice, offering the most favorable Size, Weight, Power and Cost (SWaP-C) attributes compared to other laser types. It is crucial to develop high-power, high-efficiency semiconductor laser diodes operating in the mid-infrared spectral range [11,12].

GaSb-based type-I multiple-quantum-well (MQW) diode lasers currently operate in a room-temperature continuous-wave (RTCW) regime, with emission wavelengths ranging from 1.9 to 3.5 μm [13]. Diode lasers emitting in the 1.9–2.5 μm range are capable of generating output power exceeding 1 W in RTCW operation, with wall-plug efficiencies greater than 10% from 100-μm-wide apertures [14,15]. However, the RTCW output power declines from 700 mW to 10 mW as the emission wavelength increases from 2.6 μm to 3.5 μm [16,17]. Concurrently, the threshold current density increases from below 100 A/cm^2^ to 300 A/cm^2^ per quantum well [18]. This performance degradation with increasing emission wavelength can be attributed to two primary factors. The first factor involves a reduction in the hole carrier confinement efficiency with longer emission wavelengths, particularly for wavelengths exceeding 2.5 μm [19], due to the small valence band offset using a quaternary alloy of Al_0.25_Ga_0.75_As_0.02_Sb_0.98_ lattice matched with GaSb as the quantum barrier. Insufficient hole confinement results in an undesirable increase in the density of hole states within the active region, including barrier and waveguide states with larger volumes. Thus, high injection carrier concentrations are necessary to maintain separation of the electron and hole quasi-Fermi levels, which in turn leads to an increase in the threshold current density. Additionally, the higher threshold carrier concentration amplifies non-radiative recombination currents, thereby limiting the overall laser performance. The excessive leakage of holes into the barrier and waveguide regions further exacerbates free-carrier absorption and reduces carrier injection efficiency. The second factor contributing to performance degradation is the poor crystalline quality of the InGaAsSb/AlGaInAsSb quantum wells, especially InGaAsSb, which is a consequence of the large miscibility gap in this quaternary alloy [20,21]. The majority of electrons and holes are confined in the InGaAsSb alloy, which plays the most direct role in the radiative recombination efficiency of non-equilibrium carriers. However, the poor crystalline quality promotes non-radiative recombination of carriers, which in turn reduces the internal quantum efficiency of the device and adversely affects its output characteristics. To improve the performance of GaSb-based type-I MQW lasers, it is thus essential to enhance both the hole confinement within InGaAsSb/AlGa(In)AsSb quantum wells and the crystalline quality of the InGaAsSb material.

In this paper, hole confinement was enhanced by using Al_0.2_Ga_0.55_In_0.25_As_0.24_Sb_0.76_, lattice matched with GaSb, as a quantum barrier, which provides higher valence band offsets compared to the quaternary alloy Al_0.25_Ga_0.75_As_0.02_Sb_0.98_. The calculated results indicate that the valence band offset increased from 0.102 eV to 0.166 eV, and simultaneously the conduction band offset decreased from 0.665 eV to 0.270 eV, leading to a more uniform distribution of injected carriers across multiple quantum wells. Additionally, the energy levels of the designed quantum wells were calculated for both an ideal rectangular QW profile and a curved profile after element interdiffusion over a 2.8-nm diffusion length. The operating wavelength of the laser is expected to exhibit a significant blue shift, from 0.368 eV (3.4 μm) to 0.425 eV (2.9 μm), due to interdiffusion at the quantum well interfaces, as confirmed by experimental results. The miscibility gap of the InGaAsSb alloy was systematically analyzed by calculation. These calculated results revealed that, when considering the effects of coherency strain energy, the critical temperature would be below 0 K, implying the disappearance of the miscibility gap [22]. As a result, the crystal and optical quality of the designed quantum wells were improved by using In_0.54_Ga_0.46_As_0.23_Sb_0.77_ with a compressive strain of 1.74%, thus bypassing the miscibility gap. This was confirmed by the observation of distinct satellite peaks in high-resolution X-ray diffraction (HR-XRD) curves, a smooth surface with an RMS of 0.19 nm, a well-defined interface between the quantum well and the quantum barrier, and room-temperature photoluminescence with high luminous efficiency. Furthermore, a novel epitaxial laser structure with low optical absorption loss in the p-cladding layer was designed employing Al_0.25_Ga_0.75_As_0.02_Sb_0.98_ as the waveguide, which improves the optical confinement factor in the active region, and by implementing gradient doping in the p-type cladding layer. InGaAsSb/AlGaInAsSb MQW laser diodes with these designs were successfully grown and fabricated. The maximum RTCW output power of 310 mW was achieved at an injection current of 4.6 A and an operated voltage of 1.46 V, with an emission wavelength around 2.9 μm.

## 2. Materials and Methods

In this study, material parameters used for calculations were sourced from a review article [23] and a related publication [24]. The energy levels of our designed quantum well were calculated using the 8-band k·p model considering the biaxial strain effect [25], while the refractive indices of the antimonide materials employed were determined based on the Adachi model [26]. We assume that after the quantum well structure in the active region of the device is grown, it undergoes prolonged high-temperature annealing, which causes interdiffusion of the element interfaces in the quantum well material. This diffusion process follows Fick’s second law, and the resulting element concentration distribution is symmetric. Previous studies have already conducted related theoretical and experimental studies on this problem [27,28,29,30]. The theoretical calculation method used in this paper is based on [28]. The critical temperatures of the InGaAsSb alloy were calculated based on the delta lattice parameter model [31]. Considering the effects of coherency strain energy, the critical temperatures were calculated based on [22].

All films were grown on N-type on-axis (100) GaSb substrates using a Veeco Gen-II molecular epitaxy beam (MBE) system (Veeco Instruments, Plainview, NY, USA) equipped with valved cracked cells for As_2_ and Sb_2_, as well as SUMO cells for Ga, In, and Al. In situ reflection high-energy electron diffraction (RHEED) was employed to monitor the epitaxial growth and to calibrate the growth temperature and growth rates. The growth temperatures in this study were calibrated based on the surface reconstruction transitions of the GaSb substrate. Specifically, the temperature at which the surface reconstruction of the GaSb substrate transitions from 2 × 5 to 1 × 3 is defined as Tc. Note that the substrates used in this work were double-polished n-type GaSb (001), with a doping source of Te at a concentration of approximately 1 × 10^17^ cm^−3^. The value of Tc in our study, measured using a thermocouple on the substrate, was 500 °C. Prior to each growth, surface oxides were removed at elevated temperatures, followed by deposition of a GaSb buffer layer to achieve a smoother substrate surface. During the growth process of the active region’s multiple quantum wells, the optimized growth parameters for In_0.54_Ga_0.46_As_0.23_Sb_0.77_ were as follows: growth temperature Tc, growth rates of GaSb at 0.5 ML/s and InAs at 0.587 ML/s. The optimized growth parameters for Al_0.2_Ga_0.55_In_0.25_As_0.24_Sb_0.76_ were as follows: growth temperature Tc, growth rates of GaSb at 0.5 ML/s, InAs at 0.227 ML/s and AlSb at 0.182 ML/s. To avoid long growth interruptions, two In source furnaces were used to achieve two different growth rates, thus enabling the rapid growth of the barrier and well materials. During the entire device growth process, the 365-nm thick GaSb buffer layer was grown at Tc + 110 °C, with a growth rate of 0.5 ML/s. The 2-μm thick Al_0.5_Ga_0.5_As_0.04_Sb_0.96_ layer, lattice matched with GaSb, was grown at Tc + 130 °C, with a growth rate of 0.5 ML/s for GaSb and 0.5 ML/s for AlSb. The 400-nm thick waveguide material, Al_0.25_Ga_0.75_As_0.02_Sb_0.98_, was grown at Tc + 130 °C, with a growth rate of 0.5 ML/s for GaSb and 0.167 ML/s for AlSb. Afterward, the substrate temperature was lowered to Tc in preparation for the growth of the active region’s multiple quantum wells. After the quantum wells were grown, the substrate temperature was maintained at Tc for the subsequent growth of the waveguide and cladding materials, with the same growth rates as mentioned above. Similarly, the GaSb cap layer was grown at Tc, with a growth rate of 0.5 ML/s. Based on the thickness and growth rate of the materials grown after the multiple quantum wells, it could be deduced that the multiple quantum wells had to be maintained for more than three hours at the substrate temperature of Tc. The duration of this process determines the diffusion length of the elements within the quantum wells, which affects the band structure of the quantum wells and, in turn, determines the emission wavelength of the device. Therefore, the active region of the laser, comprising multiple quantum well layers, underwent annealing at temperature Tc for a duration of up to three hours.

The crystal and structural quality of the samples were assessed using atomic force microscopy (AFM) with a Park NX20 system and high-resolution X-ray diffraction (HR-XRD) with a Bruker JV-QC3 system (Bruker, Billerica, MA, USA). Optical quality was evaluated through room-temperature photoluminescence (PL) measurements conducted using a Bruker Vertex 80 Fourier-transform infrared (FTIR) spectrometer, excited with a 1064 nm pumped laser. The TEM-ready samples were prepared using focused ion beam (FIB) FEI DB2345, yielding lamellae approximately 50 nm in thickness. Transmission electron microscopy (TEM) imaging was performed with an aberration-corrected JEOL JEM-ARM200F microscope (JEOL Ltd., Tokyo, Japan) operating at 200 kV in scanning transmission electron microscopy (STEM) mode, equipped with a high-angle annular dark-field (HAADF) detector. The grown wafer was processed into a Fabry–Pérot (FP) cavity laser with a ridge waveguide width of 100 μm, using standard contact optical lithography in combination with inductively coupled plasma (ICP) for producing the ridge waveguide and reactive-ion etching (RIE) for opening the current injection window. A 250-nm thick SiO_2_ insulation layer was deposited via plasma-enhanced chemical vapor deposition (PECVD). Low-resistance ohmic contacts on the p-type and n-type sides were achieved with magnetron sputtered 50 nm/50 nm/300 nm Ti/Pt/Au and 50 nm/300 nm AuGeNi/Au. Finally, a single emitter was chipped and attached p-side down to a copper heatsink, ready to test its performance. The heatsink temperature was controlled by the thermoelectric cooler. Output power was measured using a Coherent PowerMax PM10 sensor with a FieldMaxII meter (Coherent Inc., Saxonburg, PA, USA), and emission spectra were recorded using a Bruker Vertex 80 FTIR spectrometer equipped with a liquid nitrogen-cooled MCT detector.

## 3. Results and Discussion

### 3.1. Active Region Design

**Figure 1:**
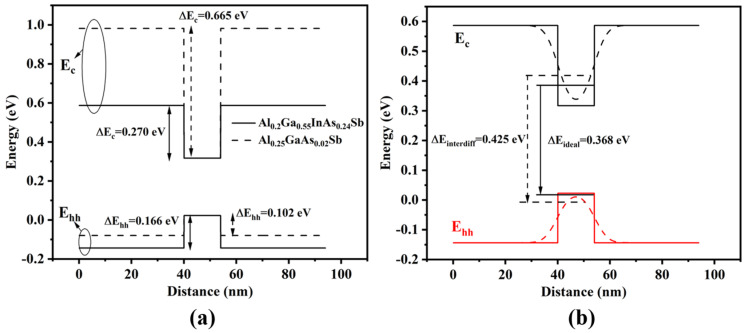
(**a**) The energy bands of the In0_.54_Ga_0.46_As_0.23_Sb_0.77_/Al_0.25_Ga_0.75_As_0.02_Sb_0.98_ and In_0.54_Ga_0.46_As_0.23_Sb_0.77_/Al_0.2_Ga_0.55_In_0.25_As_0.24_Sb_0.76_ quantum wells, (**b**) the conduction and valence band edges and the energy levels of a 14-nm In_0.54_Ga_0.46_As_0.23_Sb_0.77_/40-nm Al_0.2_Ga_0.55_In_0.25_As_0.24_Sb_0.76_ quantum well for both an ideal rectangular QW profile (solid line) and a curved profile after intermixing (dashed line) at a 2.8-nm diffusion length. The arrows mark the respective transitions between the confined conduction and valence band levels (solid line arrow for ideal rectangular QW; dashed line arrow for intermixed QW).

In this MQW design, the quinary alloy Al_0.2_Ga_0.55_In_0.25_As_0.24_Sb_0.76_ was used as the quantum barrier for the In_0.54_GaAs_0.23_Sb quantum well, enhancing hole carrier confinement compared to the quaternary alloy Al_0.25_Ga_0.75_As_0.02_Sb_0.98_. Figure 1a shows the energy bands of In_0.54_GaAs_0.23_Sb/Al_0.25_GaAs_0.02_Sb and In_0.54_GaAs_0.23_Sb/Al_0.2_Ga_0.55_InAs_0.24_Sb quantum wells, demonstrating that the valence band offset was enlarged from 0.102 eV to 0.166 eV and the conduction band offset was reduced from 0.665 eV to 0.270 eV. Strengthening the hole carrier confinement would enhance the carrier’s internal quantum efficiency, reduce optical loss, and suppress the Auger recombination current to boost the output performance of laser diodes. The proper conduction band offset would ensure a more uniform distribution of injected carriers across more than one quantum well. Figure 1b displays the conduction and valence band edges and the energy levels of our designed InGaAsSb/AlGaInAsSb quantum well, for both an ideal rectangular QW profile (solid line) and a curved profile after intermixing (dashed line) at a 2.8-nm diffusion length. This value of diffusion length was assumed by the degree of blue shift in our laser’s emission wavelength and the results in paper [29]. The arrows mark the respective transitions between the confined conduction and valence band of the first energy levels. The composition interdiffusion between the well and barrier causes a graded profile of band energy edges of QW. That makes the potential narrower at the bottom and broader at the top with respect to the ideal rectangular shape. As can be seen, the transition energy between the c1 of conduction and the hh1 of the valence level would be larger than the ideally rectangular QW (0.426 eV vs. 0.368 eV). This suggests the lasing wavelength of our designed laser would experience a blue shift from 3.4 μm to 2.9 μm when the MQW are annealed at a high temperature with the hypothesis that the diffusion length is 2.8 nm.

**Figure 2:**
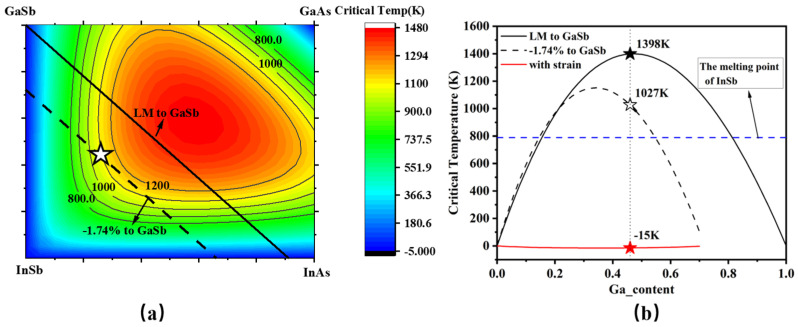
(**a**) Calculated critical temperatures for InGaAsSb neglecting the effects of coherency strain energy. The white star refers to the critical temperature of In_0.54_Ga_0.46_As_0.23_Sb_0.77_. (**b**) Calculated critical temperatures for InGaAsSb lattice matched to GaSb and with a compressive strain of 1.74% to GaSb, with and without considering the effects of coherency strain energy. The melting point of InSb is 788 K.

In addition, there is a large and severe thermodynamic miscibility gap in the quaternary alloy of InGaAsSb, which causes spinodal decomposition and phase separation, bringing many challenges in optimizing the growth of high-quality materials [32]. Relevant studies [22,31] have already introduced the critical temperature parameter for quantitative theoretical research on the miscibility gap issue in multicomponent alloys. When the actual growth temperature is below the critical temperature, the material would experience compositional segregation or phase separation due to the miscibility gap. On the other hand, when the actual growth temperature exceeds the critical temperature, no miscibility gap problem occurs. Moreover, prior studies have indicated that introducing appropriate strain into InGaAsSb can effectively suppress spinodal decomposition, thereby enhancing the crystal quality [22]. Therefore, the critical temperature of InGaAsSb was calculated to determine the optimal indium and arsenic compositions to mitigate spinodal decomposition. Spinodal decomposition is expected to be eliminated when the growth temperature exceeds the critical temperature. Figure 2a illustrates the two-dimensional distribution of critical temperatures for InGaAsSb with varying compositions, neglecting the effects of coherency strain energy. The results reveal that critical temperatures increase as indium and arsenic compositions approach 0.5, owing to the distinct properties of these elements. The maximum growth temperature for InGaAsSb is constrained to 515 °C (788 K), the melting point of InSb. Figure 2b compares the critical temperatures of InGaAsSb under three conditions: lattice-matched to GaSb, with a compressive strain of 1.74% relative to GaSb, and considering or neglecting the effects of coherency strain energy. The compositional region susceptible to spinodal decomposition is significantly reduced when a compressive strain of 1.74% is applied compared to the lattice-matched case. Furthermore, when the effects of coherency strain energy are considered, the compositional region for spinodal decomposition is effectively eliminated. Based on these findings, In_0.54_Ga_0.46_As_0.23_Sb_0.77_ was selected as the quantum well material, as it can be grown with high crystal quality while avoiding spinodal decomposition.

### 3.2. Characterization of InGaAsSb/AlGaInAsSb MQW

**Figure 3:**
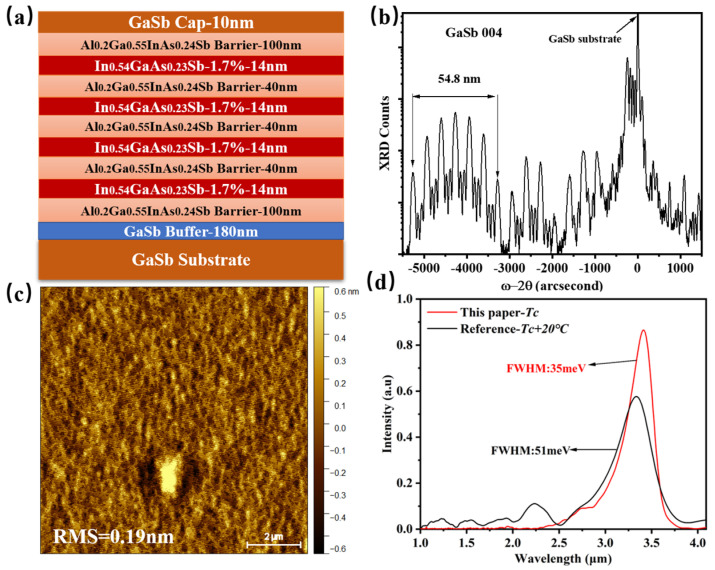
Characterization of In_0.54_Ga_0.46_As_0.23_Sb_0.77_/Al_0.2_Ga_0.55_In_0.25_As_0.24_Sb_0.76_ MQW. (**a**) The epitaxial structure of the MQW test sample. (**b**) HR-XRD ω-2θ rocking scans. (**c**) AFM image of the sample surface over 10 μm × 10 μm area. The surface RMS values are also shown in the figure. (**d**) Room temperature photoluminescence (PL) results. The red line refers to the optimized growth temperature at Tc for the active region, while the black line refers to the growth temperature at Tc + 20.

**Figure 4:**
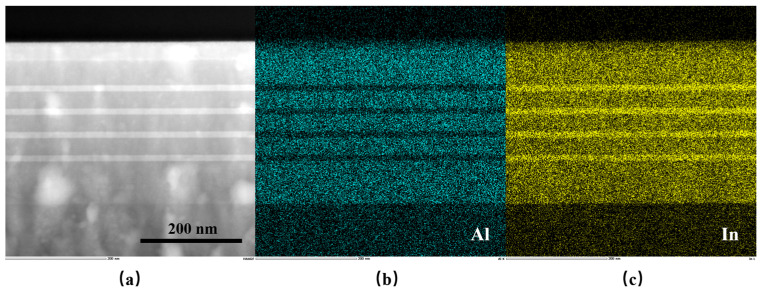
General view of the MQW sample. (**a**) HAADF image, (**b**) Al mapping, and (**c**) In mapping from EDX.

**Figure 5:**
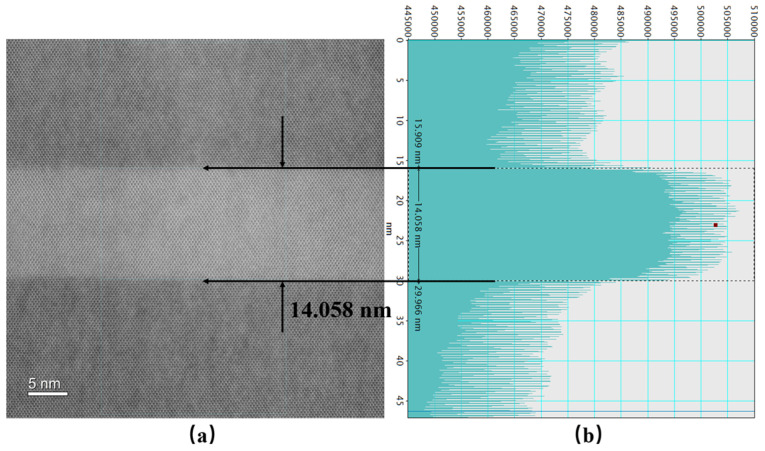
(**a**) Cross-sectional filtered HAADF-STEM images depicting a single QW of In_0.54_Ga_0.46_As_0.23_Sb_0.77_/Al_0.2_Ga_0.55_In_0.25_As_0.24_Sb_0.76_ with (**b**) the average intensity of imaged atoms along the growth direction of the material.

To confirm that our designed active region with In_0.54_Ga_0.46_As_0.23_Sb_0.77_/Al_0.2_Ga_0.55_In_0.25_As_0.24_Sb_0.76_ MQW could be grown with high crystalline and optical quality, the test sample with four quantum wells were grown and characterized by HR-XRD, AFM, and PL, as shown in Figure 3. Figure 3a displays the epitaxial structure of the test sample with four In_0.54_Ga_0.46_As_0.23_Sb_0.77_/Al_0.2_Ga_0.55_In_0.25_As_0.24_Sb_0.76_ MQW. The 100-nm Al_0.2_Ga_0.55_In_0.25_As_0.24_Sb_0.76_ layer was used to improve the photo-generated carrier confinement to enhance the luminescence efficiency. The periodicity of the quantum wells and barriers produced a series of satellite peaks on HR-XRD curves, shown in Figure 3b. The total thickness of the quantum well and quantum barrier was determined to be 54.8 nm by fitting the spacing of these satellite peaks, which is consistent with our design. The small root-mean-square value of the AFM image (0.19 nm), shown in Figure 3c, results from two-dimensional-layered growth. The clear satellite peaks and the nearly atomic flatness indicate the high crystal quality of the grown sample. Figure 3d shows the room-temperature (RT) photoluminescence (PL) results obtained from the MQW active region at growth temperatures of Tc and Tc + 20 °C. The RT PL of the sample grown at Tc displayed high PL intensity and a narrow FHWM of 35 meV, demonstrating that our designed InGaAsSb/AlGaInAsSb MQW has high luminescence efficiency compared to the sample grown at Tc + 20 °C. Additionally, more in-depth PL studies could be conducted by measuring PL under varying pump intensities and employing the ABC model for quantitative characterization of the radiative recombination mechanisms [33]. As seen in the PL spectrum, the peak wavelength is approximately 3.4 μm, corresponding to a transition energy between c1 and hh1 levels of about 0.365 eV, which closely matches our calculated result (0.368 eV) for the ideal rectangular QW profile shown in Figure 1b. Figure 4b,c presents the Al atom and In atom mappings from energy-dispersive X-ray spectroscopy (EDX) based on the HADDF image shown in Figure 4a. These mappings clearly depict the four In_0.54_Ga_0.46_As_0.23_Sb_0.77_/Al_0.2_Ga_0.55_In_0.25_As_0.24_Sb_0.76_ quantum wells with well-defined interfaces between the quantum wells and quantum barriers. Moreover, a high-magnification HAADF-STEM image of a single quantum well is shown in Figure 5a. Figure 5b shows the average intensity profile along the growth direction of the sample, as obtained using Digital Micrograph (Version 3.22.1461.0) software. This profile illustrates the well-defined interface between the quantum well and the quantum barrier. The width of the grown quantum well, measured at 14.058 nm, is consistent with our designed value. Thus, the characterization results demonstrate that the grown MQW sample has an ideal rectangular QW profile. That is reasonable because the MQW of the PL test sample was not annealed at high temperatures over a long time. However, the MQW in the entire epitaxial laser wafer underwent high-temperature annealing until the laser wafer growth was complete. Therefore, the lasing wavelength of our designed diode laser would experience a blueshift because of the composition interdiffusion, concerning the peak wavelength of this PL spectrum.

### 3.3. Laser Design and Growth

**Figure 6:**
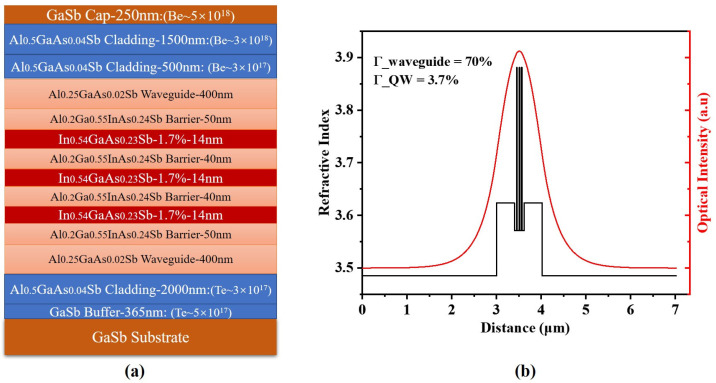
(**a**) The whole epitaxial structure of our designed GaSb-based MQW diode laser, and (**b**) Black line refers to the refractive index of the epitaxial structure and red line refers to the optical near field of fundamental mode distribution in the growth direction.

**Figure 7:**
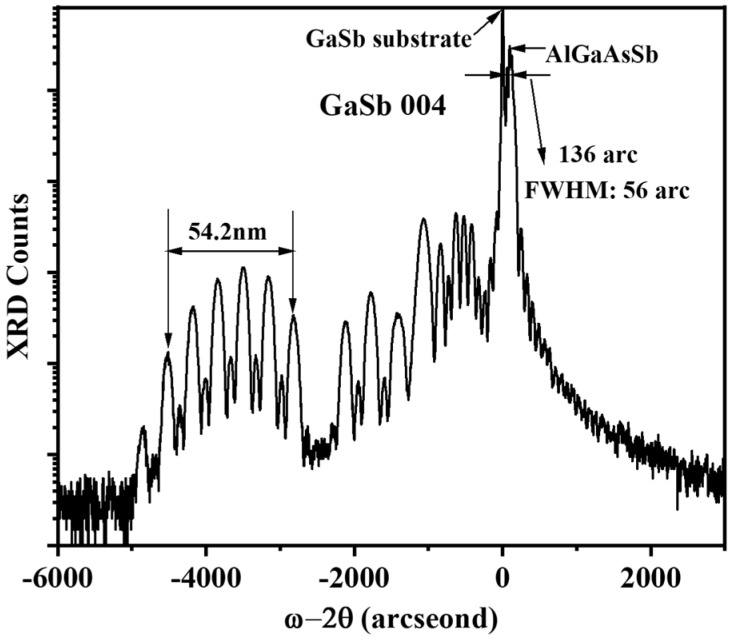
HR-XRD ω-2θ rocking scans of the grown laser wafer.

Figure 6a depicts the whole epitaxial structure of our designed GaSb-based diode laser with three 14-nm thick In_0.54_Ga_0.46_As_0.23_Sb_0.77_ quantum wells. A 40-nm thick barrier of Al_0.2_Ga_0.55_In_0.25_As_0.24_Sb_0.76_ was placed between these quantum wells to confine the carriers and suppress interaction between the quantum wells, and the MQW active region is sandwiched between a 50-nm thick Al_0.2_Ga_0.55_In_0.25_As_0.24_Sb_0.76_ layer and a 400-nm thick Al_0.25_GaAs_0.02_Sb layer. The 50-nm thick Al_0.2_Ga_0.55_In_0.25_As_0.24_Sb_0.76_ layer prevents the carriers in the quantum wells from leaking into the waveguide layer. The 400-nm thick Al_0.25_Ga_0.75_As_0.02_Sb_0.98_ layer, having a higher refractive index than Al_0.2_Ga_0.55_In_0.25_As_0.24_Sb_0.76_, is used to confine the optical field in the active region to enhance the effective optical gain and reduce the free carrier absorption loss in doped cladding layers. The refractive index of the epitaxial structure and the optical near field of fundamental mode distribution in the growth direction are shown in Figure 6b. The optical field confinement in the waveguide layer and MQW layers was calculated as 70% and 3.7%, respectively. The n-type cladding and p-type cladding layers were 2-μm thick Al_0.5_Ga_0.5_As_0.04_Sb_0.96_ doped Te and Be, respectively. Additionally, a step doping concentration profile in the 2-μm thick p-type cladding layer was used to reduce the optical loss due to hole absorption. Figure 7 presents the GaSb (004) HR-XRD characterization results from the grown laser wafer. The QW/barrier periodicity produced a series of satellite peaks. The thickness of the quantum well and quantum barrier was determined to be 54.2 nm, which is consistent with our design. The mismatch between the peaks of the GaSb substrate and AlGaAsSb is about 136 arcseconds with a FWHM of 56 arcseconds, which supports the growth of about a 6-μm thick epitaxial layer without reducing crystal quality.

### 3.4. Laser Output Performance

**Figure 8:**
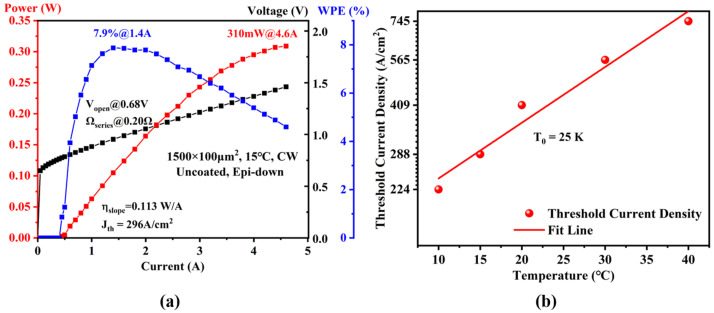
(**a**) CW output characteristics of a 1.5-mm-long and 100-μm-wide GaSb-based laser at 15 °C, and (**b**) the characteristic temperature T_0_ of this laser in CW condition.

The continuous-wave output power-current and voltage-current characteristics of the 1.5-mm-long and 100-μm-wide uncoated diode lasers, measured at a heatsink temperature of 15 °C, are displayed in Figure 8a. Note that the raw data shown in Figure 8a were based on the measured optical power from a single facet of the uncoated laser. The maximum optical power of 310 mW with an operating voltage of 1.46 V was obtained at an injection current of 4.6 A. This low-operated voltage is suitable for applications that require low energy consumption and photonic integrated circuits. The threshold current density of 296 A/cm^2^ (below 100 A/cm^2^ per QW) and a slope efficiency of 0.113 W/A were measured near the threshold current, owing to the step doping concentration profile in the p-cladding layer. Additionally, the WPE-I curve clearly shows a significant decrease in the laser’s optoelectronic conversion efficiency when the injection current exceeds 1.5 A. This degradation is primarily attributed to heat accumulation in the active region, which exacerbates non-radiative recombination processes, particularly Auger recombination. Consequently, the internal quantum efficiency decreases, leading to a reduction in the external quantum efficiency and, subsequently, a decline in slope efficiency. This phenomenon can be further investigated through detailed experimental studies using the ABC model to quantitatively analyze the variation in non-radiative recombination intensity with carrier injection levels [33]. As for voltage–current curves, the turn-on voltage of 0.68 V and the series resistance of 0.20 Ω are calculated by implementing a linear regression between the voltage and current above the threshold current. This turn-on voltage could be reduced by adding some transition layers between the GaSb and AlGaAsSb layers in our designed epitaxial laser structure. Figure 8b shows the characteristic temperature T_0_ to be 25 K, which was underestimated because the experimental data were obtained from the laser operating in CW condition. A maximum RTCW optical power of 310 mW with a threshold current density below 100A/cm^2^ per QW was obtained from a single emitter with a cavity length of 1.5 mm and uncoated facets, which outperforms a leading result among the reported results to date. The superior output performance can be attributed to the high crystalline quality and the novel waveguide design of the laser, which represent critical factors that have been inadequately addressed in previous studies [16,17].

**Figure 9:**
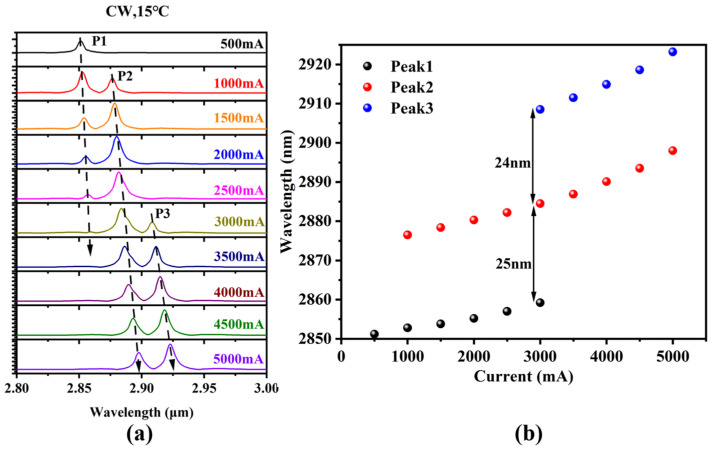
(**a**) The variation in our laser’s emission spectrum with injection current and (**b**) the peak wavelength variation of three modes with injection current.

Figure 9a displays the variation in the laser’s emission spectrum with injection current. There are three main modes with a wavelength separation of approximately 25 nm between each mode, as shown in Figure 9b, and the full width at half-maximum (FWHM) of each mode ranges from 5 to 10 nm in the output spectrum. This value of FWHM is consistent with the spectrum obtained from our 2 μm laser in our previous paper [12]. Based on relevant literature [34,35,36], these three modes might be caused by inhomogeneities in the three quantum wells of our laser, primarily due to the segregation of In atoms during the growth process. The degree of this segregation increases as the In composition is raised. The output spectrum demonstrates that our designed laser operated around 2.9 μm. The emission wavelength of our laser was precisely predicted by considering the composition interdiffusion between the well and barrier with a 2.8-nm diffusion length, as shown in Figure 1b. In this theoretical calculation, the blue shift in the laser’s emission wavelength is quantitatively explained by assuming that the diffusion lengths of In atoms, Ga atoms, and Sb atoms are all 2.8 nm. A detailed calculation model can be found in references [28,30]. Based on the above experimental observations, we speculate that the segregation of the In composition along the growth direction during the growth of the InGaAsSb multiple quantum wells leads to an uneven distribution of quantum well compositions, resulting in multiple mode peaks in the device’s emission spectrum. Additionally, the long annealing time after the growth of the multiple quantum wells causes a significant overall blue shift in the device’s emission peak. Specific and direct experimental characterization studies will be conducted in future experiments.

## 4. Conclusions

In conclusion, we demonstrated an electrically pumped RTCW mid-infrared high-power GaSb-based MQW diode laser by designing a novel epitaxial laser structure with enhancements in carrier confinement and optical field. A quinary alloy of Al_0.2_Ga_0.55_In_0.25_As_0.24_Sb_0.76_ was used as the quantum barrier to improve the hole confinement, enhancing the internal quantum efficiency from the perspective of designing the MQW active region. In the view of designing the epitaxial laser structure, Al_0.25_Ga_0.75_As_0.02_Sb_0.98_, which has a higher refractive index than Al_0.2_Ga_0.55_In_0.25_As_0.24_Sb_0.76_, was used as the waveguide layer to enhance the optical field in the active region to hold high effective optical gain and reduce the free carrier absorption loss in doped cladding layers. In addition, the p-type cladding layer used a two-step doping profile to decrease the optical absorption loss in the p-cladding layer. As for material growth, In_0.54_Ga_0.46_As_0.23_Sb_0.77_, with a compressive strain of 1.74%, was used as the quantum well to improve the crystal quality by eliminating spinodal decomposition, confirmed by HR-XRD with clear satellite peaks, a smooth surface with a root mean square of 0.19 nm, shape growth interfaces, and room-temperature photoluminescence with high luminous efficiency. A maximum RTCW optical power of 310 mW was obtained from a 1500 × 100 μm^2^ uncoated single emitter. More importantly, the lasing wavelength of our laser exhibited a significant blue shift, compared to the peak wavelength of the PL sample containing only the MQW region. This phenomenon was precisely explained by considering the composition interdiffusion between the well and barrier.

## Data Availability

The datasets used and analyzed during the current study are available from the corresponding author upon reasonable request.
